# Light behind the curtain: photoregulation of nuclear architecture and chromatin dynamics in plants

**DOI:** 10.1111/nph.14269

**Published:** 2016-11-04

**Authors:** Giorgio Perrella, Eirini Kaiserli

**Affiliations:** ^1^ Institute of Molecular, Cell and Systems BiologyCollege of Medical, Veterinary and Life SciencesUniversity of GlasgowGlasgowG12 8QQUK

**Keywords:** chromatin modifications, gene expression, light signalling, nuclear architecture, photomorphogenesis, photoreceptors

## Abstract

Light is a powerful stimulus regulating many aspects of plant development and phenotypic plasticity. Plants sense light through the action of specialized photoreceptor protein families that absorb different wavelengths and intensities of light. Recent discoveries in the area of photobiology have uncovered photoreversible changes in nuclear organization correlated with transcriptional regulation patterns that lead to de‐etiolation and photoacclimation. Novel signalling components bridging photoreceptor activation with chromatin remodelling and regulation of gene expression have been discovered. Moreover, coregulated gene loci have been shown to relocate to the nuclear periphery in response to light. The study of photoinduced changes in nuclear architecture is a flourishing area leading to major discoveries that will allow us to better understand how highly conserved mechanisms underlying genomic reprogramming are triggered by environmental and endogenous stimuli. This review aims to discuss fundamental and innovative reports demonstrating how light triggers changes in chromatin and nuclear architecture during photomorphogenesis.

## Introduction

### Light shapes plant development

Light is an energy source as well as an informational signal that influences plant architecture and optimizes plant growth. In addition to making their own food, plants utilize light as a stimulus for triggering major changes in their lifestyle to survive, grow and reproduce. Plant development is determined by the presence or absence of light. In particular, light drives one of the most life‐changing developmental transitions in plant development: photomorphogenesis or de‐etiolation, the ‘birth of body formation’, occurs as soon as a seedling emerges from soil and sees light for the first time (Kaiserli & Chory, 2016). De‐etiolation is characterized by a series of responses: the opening and greening of the cotyledons (embryonic leaves) and the inhibition of hypocotyl (embryonic stem) growth (Chen & Chory, 2011). Light quality, intensity and duration provide a huge amount of information regarding the plant's surrounding environment, such as the time of day, the time of year and the presence of competitors.

Plants are sessile organisms that are highly adaptable to changing environmental conditions and resource availability. Changes in plant body structure, physiology and metabolism lead to maximal light capture and optimal growth. Evolution has led to a highly sophisticated suite of plant photoreceptors that sense the quantity, spectral quality, direction and periodicity of light. In Arabidopsis, there are five distinct families of photoreceptors: UV‐RESISTANCE LOCUS 8 (UVR8), the first genetically encoded UV‐B receptor; the blue light receptors cryptochromes 1 and 2 (cry1 and cry2); phototropins 1 and 2 (phot1 and phot2); the red (R)/far‐red (FR) light sensors phytochromes A–E (phyA–E); and the Zeitlupe family of clock proteins (ZEITLUPE, FLAVIN‐BINDING, KELCH REPEAT, F‐BOX 1, LOV KELCH PROTEIN2) (Wu, 2014). Communication between different light signalling pathways is achieved by antagonistic or synergistic crosstalk among these five distinct photoreceptor families. Signal integration during the early stages of photomorphogenesis occurs primarily at the level of gene expression. Transcription factors (TFs) and transcriptional regulators interact with photoreceptors and light signalling components to induce major developmental programming by activating or repressing the transcription of key light‐responsive genes. Global transcriptomic analysis shows that light regulates the abundance of thousands of transcripts, reaching *c*. 70% of the Arabidopsis genome (Tepperman *et al*., 2001; Jiao *et al*., 2007). Transcriptional regulation of gene expression occurs through the action of multiple protein complexes (activators, repressors, remodelling enzymes, adaptors, polymerases) as well as the deposition of chemical modifications on histones and DNA itself (Macrae & Long, 2012). Here, we introduce how various histone modifications can activate or repress transcription and focus on the role of chromatin in regulating light‐responsive genes during the early stages of photomorphogenesis.

## Histones organize and remodel the genome

The basic unit of the nucleosome is an octamer composed of two copies of histones (H) H2A, H2B, H3 and H4 around which 146 bp of DNA are wrapped (Arya & Schlick, 2009; Zhou *et al*., 2013). Specific histone variants such as H2A.Z, H3.3 and CenH3 are recruited to nucleosomes to regulate gene expression and genome structure in response to endogenous, environmental stimuli and developmental stages (Deal *et al*., 2007; Bernatavichute *et al*., 2008; Yan *et al*., 2010; Coleman‐Derr & Zilberman, 2012). In eukaryotes, the N‐terminal histone tails as well as the core histone domains that are enriched in basic amino acids such as lysine (K) and arginine (R) can be reversibly modified by the addition of different moieties as a means of altering DNA accessibility.

One of the most important and dynamic features of euchromatin (lightly packed and more readily accessible DNA) and heterochromatin is the diversity of post‐translational modifications of nucleosomal histones, also referred to as the ‘histone code’ (Jenuwein & Allis, 2001). The complexity of the histone code is attributed to the combination of single or multiple chemical modifications, ranging from methylation, acetylation to phosphorylation, ubiquitination, sumoylation and ADP ribosylation of key lysine and, to a lesser extent, arginine residues primarily located at the N‐terminal histone tails. Such modifications have been linked to the regulation of different types of fundamental nuclear processes such as DNA replication, transcription, repair and chromatin condensation (Kouzarides, 2007). In addition to histone tail methylation, DNA can also become a target for chemical modifications. In particular, DNA methylation of cytosines (C) in a symmetric or asymmetric context (CG, CHG or CHH) is particularly distributed in chromosomal areas rich in transposable elements (TEs) and in pericentromeric regions (Finnegan *et al*., 1998). DNA methylation leads to a higher degree of chromatin compaction (heterochromatin), therefore preventing access of the transcriptional machinery to DNA, which leads to silencing of TEs and any genes in the vicinity (Tariq & Paszkowski, 2004).

Remodelling of nucleosomes can occur either via movements of the histone octamer or by altering the nucleosomal composition. Specialized ATP‐dependent enzymes alter the position of nucleosomes, thereby modulating the accessibility to DNA (Narlikar *et al*., 2013). Chromosomal remodelling can also occur by exchanging canonical histone units with histone variants (Rando & Ahmad, 2007).

Plants contain the following histone variants: H3.1, H3.3, H2AX and H2AZ (Talbert & Henikoff, 2010). In addition, H2AW was identified as a novel plant‐specific H2A variant and acts as a major player in heterochromatin silencing (Talbert & Henikoff, 2010; Yelagandula *et al*., 2014). By contrast, H3.3 and H2AZ are primarily involved in active transcription. Very elegant studies have shown preferential deposition of H2AZ at the first nucleosome after a transcriptional start site (TSS) and H3.3 enrichment in promoter regions and gene bodies (Zilberman *et al*., 2008; Shu *et al*., 2014).

## Chromatin remodelling and light signaling

Dynamic changes in chromatin structure and architecture through large‐scale genome reorganization or localized modifications on histone tails provide excellent tools for conferring specificity to gene expression in response to environmental and endogenous stimuli at diverse tissues and at different developmental stages (Kouzarides, 2007; Barneche *et al*., 2014). Light has direct and indirect roles in mediating histone modifications and chromatin reorganization as a means of regulating gene expression to trigger major developmental transitions, ranging from de‐etiolation to shade avoidance and flowering (Kaiserli & Chory, 2016). Early studies demonstrating a direct association between one of the major light signalling components, DE‐ETIOLATED‐1 (DET1), with histones (H2B) provided evidence supporting the involvement of chromatin remodelling in light signalling (Benvenuto *et al*., 2002). DET1 is one of the first identified repressors of photomorphogenesis which was later shown to act via interactions with DAMAGED‐SPECIFIC DNA‐BINDING PROTEIN1 (DDB1), CONSTITUTIVE PHOTOMORPHOGENIC10 (COP10) and PHYTOCHROME INTERACTING FACTORS (PIFs) (Chory & Peto, 1990; Pepper *et al*., 1994; Schroeder *et al*., 2002; Dong *et al*., 2014). This review focuses on how light triggers specific histone modifications and global changes in chromatin architecture during photomorphogenesis and how these events lead to transcriptional and physiological outputs.

## Histone acetylation in light signalling

Mass spectrometry analysis has revealed a high degree of conservation in the position and post‐translational modification of different histone isoforms in plants and mammalian systems (Zhang & Reinberg, 2001; Earley *et al*., 2007). More specifically, each H3 and H4 tail contains six lysine residues that are acetylated on H3 (K9, K14, K18, K23, K27, K56) and five on H4 (K5, K8, K12, K16, K20). Overall, histone acetylation causes a change of charge and reduces the affinity between DNA and the nucleosomes, therefore allowing TFs to access specific DNA sequences and enhance gene expression (Kuo *et al*., 1998; Zhang *et al*., 1998; Shahbazian & Grunstein, 2007). By contrast, removing the acetyl group (deacetylation) leads to a tighter interaction between DNA and histones, causing gene repression and silencing (Kadosh & Struhl, 1998; Rundlett *et al*., 1998; Chen & Wu, 2010). Fine‐tuning the accessibility of chromatin to TFs is achieved by modulating the total amount of histone acetylation. Most plant species (Arabidopsis, tomato, maize, rice, barley and grapevine and brassica) have dedicated histone‐modifying enzymes, such as histone acetyltransferases (HATs) and histone deacetylases (HDACs), that specialize in catalysing the deposition or removal of acetyl groups on specific histones (Pandey *et al*., 2002; Chen & Wu, 2010; Papaefthimiou *et al*., 2010; Pontvianne *et al*., 2010; Aquea *et al*., 2011; Aiese Cigliano *et al*., 2013).

### Histone acetylation regulating tissue‐specific induction of PetE in response to light

One of the first examples of light‐regulated chromatin modifications, in particular histone acetylation, was found to control the tissue‐specific induction of the plastocyanin gene *PetE* in green pea (Chua *et al*., 2003). More specifically, the authors showed a light‐dependent enrichment in the acetylation pattern of H3 and H4 at the enhancer and promoter regions of *PetE* locus specifically in plant shoots (Chua *et al*., 2001). Subsequent studies monitoring the expression of the *GUS* reporter gene driven by the *PetE* promoter in transgenic tobacco plants demonstrated a link between the transcriptional activity and hyper‐acetylation based on HDAC inhibitor treatments (TSA and sodium butyrate) (Chua *et al*., 2003). Immunoprecipitation of plant chromatin using antibodies recognizing acetylated histone tails indicated an increase in acetylation of H3 and H4, respectively.

In plants, the enzymes responsible for depositing acetyl groups on histone tails, HATS, are divided into four classes: GNAT (GCN5‐related N‐terminal acetyltransferases); MYST (whose members can also acetylate non histone proteins); p300/CREB‐binding protein (CPB) (involved in cell cycle and apoptosis); and TATA binding protein‐associated factors (TAFs) (Sterner & Berger, 2000; Pandey *et al*., 2002). In Arabidopsis, *HAF2* encodes a member of the TAF1 protein complex. Chromatin immunoprecipitation (ChIP) experiments showed that *haf* mutants exhibit lower Chloroplast (Chl) accumulation owing to a reduction in H3 acetylation and a decrease in the expression of light‐responsive genes *RBCS* and *CAB2* (Supporting Information Table S1) (Bertrand *et al*., 2005).

Genetic studies on the HAT mutant, *gcn5*‐1, showed an elongated hypocotyl phenotype in response to FR light, whereas mutant plants for the HDAC HD1 (HDA19) exhibited the opposite phenotype (Benhamed *et al*., 2006). The *gcn5‐1/hd1* double mutant restored hypocotyl elongation to wild‐type values, suggesting an antagonistic action between GCN5 and HD1 (Benhamed *et al*., 2006). Similarly to *haf2*,* RBCS* and *CAB2* genes were also shown to be down‐regulated in *gcn5‐1*. However, the expression of an additional light‐regulated and INDOLE‐3‐ACETIC ACID INDUCIBLE (IAA) gene, *IAA3*, was reduced in *gcn5‐*1 and *hd1*, but not in *haf2*, indicating only partial overlapping functions between the two classes of HATs, GCN5 and TAF1. ChIP analysis using antibodies against different acetylated residues (H3K9, H3K14, H3K27, H4K5, H4K8, H4K12 and H4K16) showed that GCN5 is required to predominantly acetylate H3 residues. Analysis of the promoter elements and genes associated with GCN5 showed a clear overlap with binding targets of ELONGATED HYPOCOTYL 5 (HY5), a major positive transcriptional regulator of photomorphogenesis (Lee *et al*., 2007).

Overall, HATs and HDACs possess opposite roles: HATS such as GCN5 function as activators, whereas HDACs such as HDA19, are able to repress gene expression in response to different light stimuli (Barneche *et al*., 2014). In addition to HDA19, HDA15 has been shown to repress Chl biosynthesis in etiolated seedlings (Liu *et al*., 2013). HDA15 physically interacts with the PHYTOCHROME INTERACTING FACTOR3 (PIF3) TF in darkness. ChIP analysis showed an increase in H4 acetylation in the vicinity of photosynthetic gene loci such as *GUN5*,* LHCB2.2*,* PSBQ* and *PSAE1* in *hda15*,* pif3* single and double mutants (Liu *et al*., 2013). Detailed phenotypic analyses also showed that *hda15* hypocotyls were relatively longer than wild‐type under R and FR light conditions. Taken together, these data suggest that HDA15 and HDA19 might play an antagonistic role during hypocotyl development (Liu *et al*., 2013, 2014).

Histone deacetylases are known to be recruited by transcriptional corepressors such as TOPLESS (TPL) to regulate flower and seedling development, flowering time, circadian rhythms and hormone signalling (Krogan & Long, 2009; Krogan *et al*., 2012; Wang *et al*., 2013; Oh *et al*., 2014; Ryu *et al*., 2014; Graeff *et al*., 2016). Transcriptional corepressors are commonly associated with DNA‐binding proteins that contain repressive motifs, such as the plant‐specific ethylene‐responsive element binding factor‐associated amphiphilic repression (EAR) motif (Kagale & Rozwadowski, 2011). Such multiprotein complexes bridging transcriptional repressors with chromatin modifying and remodelling enzymes lead to epigenetic regulation of gene expression and are highly conserved in eukaryotes (Thiel *et al*., 2004; Kagale & Rozwadowski, 2011). Functional and genetic analysis and tissue‐specific composition of histone deacetylation complexes at a given developmental stage would be essential in order to understand the role of transcriptional corepressor–HDAC protein complexes in regulating gene expression during photomorphogenesis in plants.

### Histone deacetylation regulates light‐dependent changes in PHYA transcript abundance

**Figure 1:**
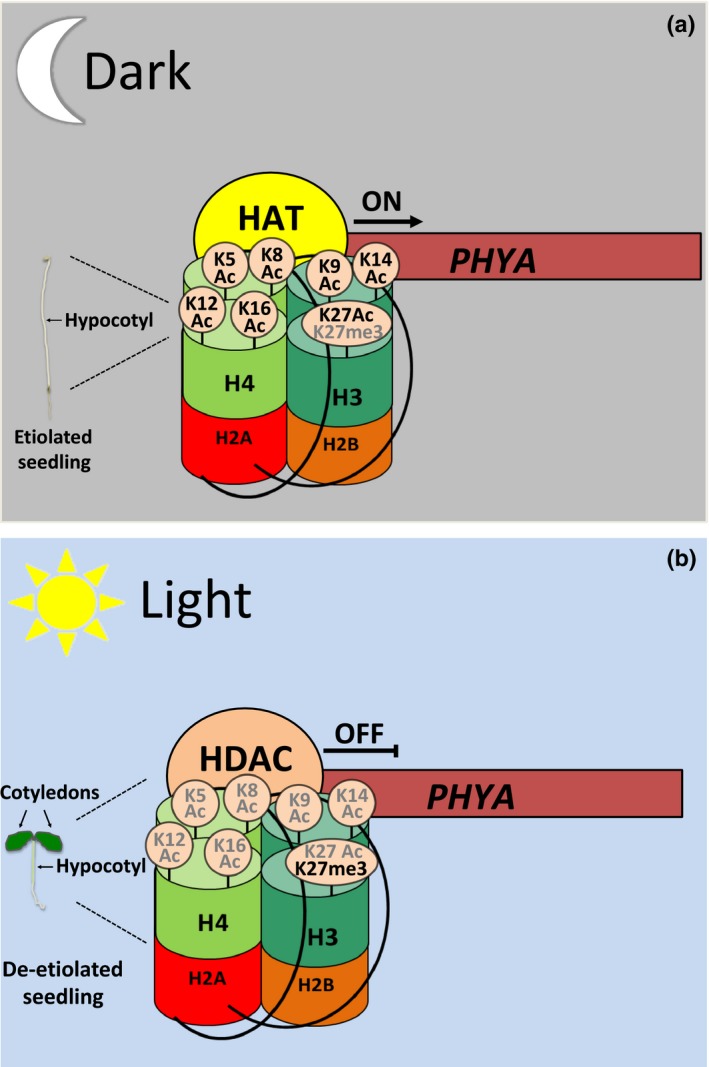
Reversible histone acetylation regulates *PHYTOCHROME A* expression during de‐etiolation. (a) Acetylation of H3 and H4 histone tails in the vicinity of the *PHYA* promoter allow its gene expression in darkness. HAT, histone acetyltransferase. ON indicates active gene expression. (b) Light exposure induces a decrease in histone acetylation and an increase in H3K27 methylation, resulting in reduced *PHYA* transcript abundance. HDAC, histone deacetylase. OFF indicates inactive gene expression. Histone modifications in bold or grey modulate gene transcription ON and OFF, respectively. The model is based on data shown in Jang *et al*. (2011).

Phytochrome A is the primary photoreceptor for FR light perception (Chen & Chory, 2011). PhyA protein accumulates in darkness in the inactive but stable Pr form (Pr). Upon FR light exposure, phyA is converted to the active Pfr form (Pfr), which is rapidly degraded by the proteasome via interactions with the E3 ubiquitin ligase, COP1 (Sharrock & Clack, 2002; Seo *et al*., 2004). At the transcript level, *PHYA* gene expression is strongly repressed by both FR and R light (Canton & Quail, 1999). In adult *Arabidopsis* plants, *PHYA* transcript abundance can be induced when plants are kept in the dark, also known as dark‐adaptation. Recent studies have revealed that changes in *PHYA* expression are accompanied by changes in histone acetylation at multiple residues: H3K9/K14 K27 as well as at H4K5, K8, K12 and K16 (Fig. 1a) (Table S1) (Jang *et al*., 2011).

Upon light exposure, the amount of histone acetylation near the *PHYA* promoter is diminished, whereas trimethylation of H3K27 is increased, indicating repression (Fig. 1a,b). The increase in acetylation during darkness is specific to promoter regions and the TSS of *PHYA* (Jang *et al*., 2011). *Hd1* mutants abolished the FR light‐dependent repression of *PHYA*, whereas H3K9/14 acetylation was maintained even after 8 h of light exposure, contrary to what has been observed in wild‐type plants where the H3 and H4 acetylation was reduced by 50% after light exposure (Jang *et al*., 2011) (Fig. 1b). Collectively these studies demonstrate that HDA19 regulates *PHYA* gene expression by inducing the deacetylation of the *PHYA* promoter region in response to light (Fig. 1).

Additional evidence for the role of light in the deposition of histone acetylation marks as a means of triggering changes in gene expression comes from time‐course experiments showing a positive correlation between an increase in white and R light‐dependent gene expression and H3K9 acetylation (Guo *et al*., 2008). The same study showed that *hy5* mutants exhibited impaired H3K9 acetylation, whereas *hd1*,* det1* and *cop1* showed augmented H3K9ac levels (Guo *et al*., 2008).

### Histone acetylation controls UV‐B photoprotective and photomorphogenic responses

UV‐B triggers transcriptional changes, the majority of which are regulated by the UV‐B receptor UVR8 (Ulm *et al*., 2004; Brown *et al*., 2005; Heijde & Ulm, 2012). UVR8 does not possess a canonical DNA‐binding domain or nuclear localization signal; however, it functions in the nucleus via a UV‐B‐dependent interaction with COP1 and therefore allows the accumulation of HY5, the main transcription factor that regulates the expression of UVR8‐dependent genes (Ulm *et al*., 2004; Kaiserli & Jenkins, 2007; Favory *et al*., 2009; Yin *et al*., 2016). In addition to its interaction with COP1, *in vitro* experiments have shown that UVR8 can associate with chromatin via histone binding (Cloix & Jenkins, 2008). ChIP studies have reported that UVR8 can associate with the promoters and gene bodies of UVR8‐regulated genes (Brown *et al*., 2005; Kaiserli & Jenkins, 2007; Cloix & Jenkins, 2008; Cloix *et al*., 2012). The exact mechanism of action and physiological significance of this association require further investigation. However, the fact that the association of UVR8 with chromatin is constitutive and not regulated by UV‐B would suggest that UVR8 could potentially enhance the recruitment of chromatin‐modifying enzymes and transcriptional regulators (Cloix & Jenkins, 2008; Cloix *et al*., 2012). There is increasing evidence supporting the role of histone modifications and, in particular, acetylation of H3K9 and H3K14 in regulating UV‐B‐mediated changes in gene expression (Cloix & Jenkins, 2008). More specifically, ChIP analysis demonstrated an enrichment of H3K9/K14ac levels on the promoters of early UV‐B‐responsive genes (*EARLY LIGHT‐INDUCABLE PROTEIN 1*,* HY5*,* HYH*), which clearly correlates with UV‐B‐dependent induction of the aforementioned genes (Brown *et al*., 2005; Cloix & Jenkins, 2008). Further evidence comes from a very recent report where ChIP sequencing analysis revealed that genome‐wide UV‐B‐mediated enrichment of H3K9 and H3K14 diacetylation depends on UVR8 (Velanis *et al*., 2016). More importantly, 40% of the identified loci showing UV‐B dependent enrichment are regulated by UVR8 (*ELIP1*,* CHS*,* HYH*,* PHR1*) (Brown *et al*., 2005; Favory *et al*., 2009; Velanis *et al*., 2016). The role of UVR8 in mediating histone modifications seems to be specific to acetylation, as the absence of UVR8 had no effect on the levels of H3K4me3, H3K9me3, H3K36me3 or H2Bub (Table S1) (Velanis *et al*., 2016). Furthermore, pharmacological studies using an inhibitor of histone acetylation blocked the induction of UVR8‐regulated genes (Velanis *et al*., 2016). The HATs or HDACs regulating the UVR8‐dependent enrichment of H3K9/K14 diacetylation remain to be identified and the role of UVR8 in facilitating this process requires further investigation.

Consistent with findings in Arabidopsis, studies in maize have reported that UV‐B induced chromatin changes are required for transcriptional regulation of gene expression (Casati *et al*., 2006, 2008). More specifically, UV‐B‐tolerant lines exhibit greater acetylation on N‐terminal tails of histones H3 and H4 after irradiation. These acetylated histones were enriched in the promoters and transcribed regions of the UV‐B‐dependent up‐regulated genes. More recent studies in maize report that UV‐B affects H3K9 and H3K27 methylation on the promoter of *P1*, an R2R3‐MYB transcription factor that regulates the accumulation of flavonoids (Table S1) (Rius *et al*., 2016). These reports would suggest that a highly conserved UV‐B‐mediated epigenetic mechanism operates in cereals; however, whether the action of UVR8 is indispensable for this response remains to be uncovered.

## Histone methylation and photomorphogenesis

Histone methylation takes place on lysine or arginine residues and it can result in the addition of one up to three methyl groups. Methylation marks are dynamically established by histone methyltransferases (HMTs) and removed by demethylases, which are specific to a particular lysine or arginine residue (Liu *et al*., 2010; Lu *et al*., 2011). In plants, histone methylation is associated with gene activation or repression depending on the position and number of methyl groups of the mark. H3K4me3, H3K9me3 and H3K36me3 correlate with active transcription, while genes presenting H3K27me3 marks tend to have low transcript abundance (Zhang *et al*., 2007, 2009; Roudier *et al*., 2011). H3K9me2 and H3K27me1 are usually located on centromeric regions of the chromosomes and are common features of silent transposons or DNA repeats that correlate with highly methylated DNA (Bernatavichute *et al*., 2008; Zhang *et al*., 2009; Roudier *et al*., 2011).

In Arabidopsis, the HMT SGD8 (SET DOMAIN GROUP 8) regulates H3K36 methylation abundance in gene bodies (Li *et al*., 2015). Whole genome transcriptome and methylome analysis showed that SGD8 regulates the expression of light and carbon fixation‐related genes, some of the promoters of which showed overrepresentation of light‐responsive elements (LREs). Gene annotation studies and epigenome analysis revealed a correlation between SDG8‐mediated H3K36me3 deposition and activation of gene expression (Table S1) (Li *et al*., 2015). Whether SGD8 enhances the recruitment of TFs (PIFs, HY5) to LREs of those genes in response to light and circadian rhythms requires further investigation.

Studies on the distribution of histone modifications of light vs dark‐grown Arabidopsis seedlings have shown that H3K9ac and H3K27ac acetylation is more prominent in gene‐specific regions, whereas H3K9me3 and H3K27me3 are diffused in genes and, to a lesser extent, intergenic regions and TEs (Table S1). Further analysis showed that H3K27me3, unlike H3K9Ac, H3K9me3 and H3K27Ac, marked targets in a tissue‐specific manner (Charron *et al*., 2009). Analysis of the effect of the histone modifications on metabolism revealed that some pathways (i.e. photosynthesis) were mostly targeted by acetylation whereas others (i.e. GA metabolism) mostly contained H3K27me3 (Charron *et al*., 2009). Taken together, these data suggest that the transition from dark to light coordinates changes in histone modifications and transcription during seedling development. Further investigation could provide a more detailed understanding on the role of histone acetylation vs methylation in de‐etiolation.

### Histone methylation regulates phytochrome‐mediated seed germination

**Figure 2:**
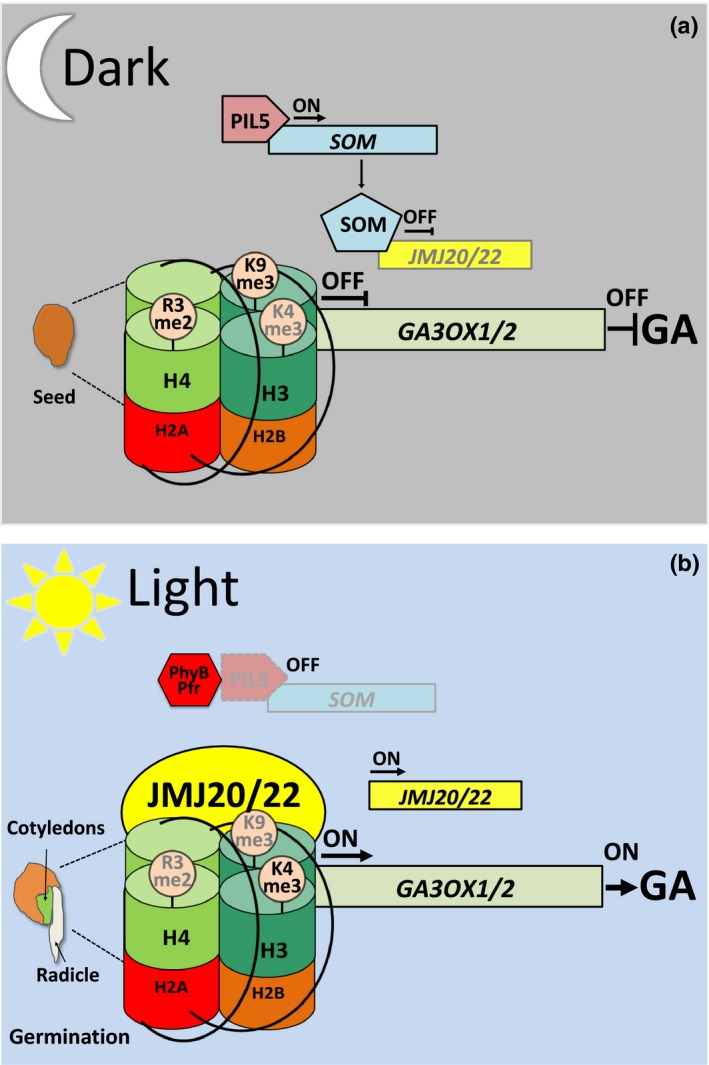
The role of the histone demethylases JMJ20/22 during phytochrome B (phyB)‐dependent seed germination. (a) Dark allows the accumulation of PIL5 that induces the expression of SOMNUS (SOM), which acts as a negative regulator of *JMJ20* and *JMJ22* expression. As a result, the levels of H3K9 and H4R3 methylation increase, leading to insufficient amounts of GA hormone production. (b) Upon light illumination, photoactivated phyB leads to a reduction of PIL5 protein. As a result, *JMJ20* and *JMJ22* are relieved from SOM‐dependent repression and induce H3K9 and H4R3 demethylation on *GA3OX1/2*, which leads to *GA3OX1/2* expression, GA production and seed germination. ON and OFF indicate active or inactive gene expression, respectively. JMJ, Jumonji C (JmjC) domain‐containing protein; PIL5, PIF3‐like 5; GA3OX1/2, GIBBERELLIN 3‐BETA‐DIOXYGENASE 1/2. Histone modifications in bold or grey modulate gene transcription ON and OFF, respectively. The model is based on data shown in Cho *et al*. (2012).

In addition to its role in photomorphogenesis, shade avoidance and photoperiodic flowering, the R light receptor phyB plays an important role in photoreversible seed germination (Shinomura *et al*., 1994). In order to allow germination, the balance between plant hormones ABA and GA plays a major role, with the former blocking and the latter favouring the process (Koornneef *et al*., 2002; North *et al*., 2010). Double mutants of the histone arginine (HR) demethylases, Jumonji C (JmjC) domain‐containing proteins, *JMJ20* and *JMJ22*, exhibited reduced phyB‐mediated seed germination in response to R light (Cho *et al*., 2012). Gene expression analysis showed R light‐dependent reduction of *GIBBERELLIN 3‐BETA‐DIOXYGENASE* 1 and 2 (*GA3OX1*,* GA3OX2)* in *jmj20/jmj22* compared with the wild‐type. Moreover, ChIP experiments showed the ability of both demethylases to bind the promoters of *GA3OX1* and *GA3OX2* (Cho *et al*., 2012). In the absence of R light stimulation when phyB is in the ground state (Pr), *JMJ20* and *JMJ22* are repressed by the zinc‐finger protein SOMNUS. The phytochrome interacting factor PIL5 (PIF3‐like) directly activates the expression of *SOMNUS* in the dark (Kim *et al*., 2008). Upon R light illumination, photoactivated phyB (Pfr) targets PIL5 for proteasomal‐mediated degradation, leading to an increase in *JMJ20* and *JMJ22* expression (Oh *et al*., 2006). As a result, the HR demethylases JMJ20 and JMJ22 reduce the levels of H4R3me2, which leads to the activation of the GA pathway to promote seed germination (Table S1; Fig. 2) (Cho *et al*., 2012).

### Light and hormone signalling coregulate histone methylation during de‐etiolation

Along with the aforementioned HMTs and demethylases, chromatin remodelling factors have also been reported to indirectly regulate the methylation status of histones in darkness. More specifically, the negative regulator of photomorphogenesis, PICKLE (PKL), belongs to the ATP‐dependent SWITCH/SUCROSE NONFERMENTING (SWI/SNF) family of chromatin remodelling factors (Ogas *et al*., 1999). Molecular, genetic and phenotypic characterization revealed that PKL functions as a repressor of light signalling by negatively regulating the trimethylation status of H3K27me3 in the vicinity of genes involved in hypocotyl elongation (Jing *et al*., 2013). Under dark conditions, PKL was shown to interact directly with the bZIP TF HY5 on the promoters of *IAA19* and *EXPANSIN2 (EXP2)* (Jing *et al*., 2013). Once recruited to chromatin, PKL antagonizes the activity of HY5 by repressing H3K27me3 deposition and therefore allowing hypocotyl elongation to proceed, which is a feature of seedling development in the dark (also referred to as skotomorphogenesis).

Recent studies have revealed that PKL stands at the crossroads of brassinosteroid (BR), GA and light signalling pathways via direct interactions with key protein components. In the absence of light, PKL physically interacts with the positive regulators of hypocotyl elongation, PIF3 and BRASSINAZOLE RESISTANT1 (BZR1) TFs, and represses the deposition of H3K27me3 marks to allow the expression of cell‐elongation genes (Zhang *et al*., 2014; Qiu *et al*., 2015). Furthermore, the GA‐sensitive growth‐repressing DELLA proteins interact and negatively regulate PKL, possibly by interfering with PIF3 binding. Exogenous application of the growth‐promoting hormones brassinolide or GA resulted in a PKL‐dependent reduction in H3K27me3 levels on cell elongation‐related genes, whereas inhibitors of BR and GA signalling led to the opposite effect (Table S1) (Zhang *et al*., 2014). These observations clearly demonstrate that PKL acts as the integrating factor regulating H3K7me3 levels in response to light, BR and GA to control hypocotyl elongation during etiolation.

## Histone monoubiquitination triggers de‐etiolation

On top of acetylation and methylation, histones can covalently and reversibly associate with larger moieties, such as ubiquitin, which is more commonly associated with targeting nonhistone proteins for proteasomal degradation (van Nocker & Vierstra, 1993; Strahl & Allis, 2000). Studies in yeast and humans have revealed that H2B monoubiquitination regulates major nuclear processes, ranging from DNA damage repair and regulation of gene expression (transcriptional initiation, elongation, mRNA processing) to nucleosomal positioning (Pavri *et al*., 2006; Moyal *et al*., 2011; Roudier *et al*., 2011; Jung *et al*., 2012). H2B monoubiquitination in yeast provides a great example of ‘histone crosstalk’ as it can act as a prerequisite for H3K4 and H3K79 mono, di and trimethylation, leading to repression or activation of gene expression, respectively (Latham & Dent, 2007). A novel approach using photocrosslinking technology has provided evidence on the molecular mechanism by which monoubiquitinated H2B recruits and ‘corrals’ the human methyltransferase Dot1 into an enzymatically active orientation in order to methylate H3K79 (Zhou *et al*., 2015).

**Figure 3:**
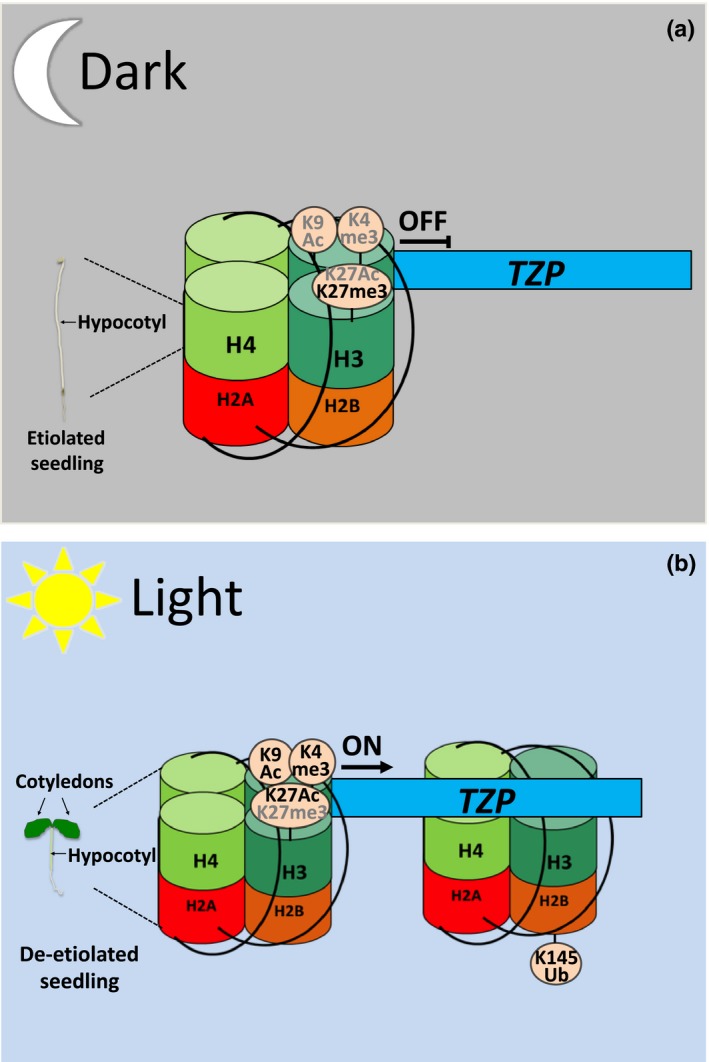
A model for the role of H2B monoubiquitination in de‐etiolation. (a) Chromatin modifications such as H3K27 trimethylation keep light‐induced loci under tight control in darkness. (b) During de‐etiolation, an increase in H3K4/K27 acetylation and H3K4 methylation induce the initiation of light‐regulated gene expression, such as *TZP*. H2BK145 monoubiquitination on the *TZP* gene body is proposed to promote transcriptional elongation. TZP, tandem zinc‐knuckle PLUS3. ON and OFF indicate active or inactive gene expression, respectively. Arrows indicate initiation of transcription. Histone modifications in bold or grey modulate gene transcription ON and OFF, respectively. The model is based on data shown in Bourbousse *et al*. (2012).

In Arabidopsis, mass spectrometry analysis revealed that the plant histone H2B isoform can become monoubiquitinated on K145 (Bergmuller *et al*., 2007). Very elegant studies have recently established an active role of H2BK145ub in de‐etiolation, one of the most dramatic developmental transitions during the life cycle of a plant (Bourbousse *et al*., 2012). The absence of the main enzyme responsible for H2B monoubiquitination in *hub1‐3* mutant plants leads to impaired de‐etiolation and slower kinetics of light‐regulated genes involved in Chl biosynthesis such as *LHCA1* and *GUN5* or signal integration (Bourbousse *et al*., 2012). ChIP‐chip analysis revealed that a 6 h light exposure triggers an increase in H2B monoubiquitination in the body of 272 genes, the majority of which are up‐regulated (Bourbousse *et al*., 2012) (Fig. 3). Furthermore, the authors showed a correlation between H2BK145ub deposition and light‐dependent H3K4me3 and H3K36me3 enrichment on gene loci coding for major light signalling integrating components such as SUPPRESSOR OF PHYTOCHROME A 1 (SPA1), TANDEM ZINC‐KNUCKLE PLUS3 (TZP) and GIGANTEA (GI) (Table S1; Fig. 3) (Hoecker *et al*., 1998; Huq *et al*., 2000; Loudet *et al*., 2008; Bourbousse *et al*., 2012; Kaiserli *et al*., 2015).

However, it remains to be established whether H2B monoubiquitination can directly influence the methylation status of histones in a similar way to yeast. No direct correlation was observed between the levels of H2BK145ub and the light‐dependent repression or down‐regulation of gene expression (Bourbousse *et al*., 2012). A cumulative deposition of the H2BK145ub histone mark could act as a rheostat for modulating rapid changes in the expression of light‐ and circadian‐regulated genes to optimize plant growth (Table S1; Fig. 3) (Bourbousse *et al*., 2012) Whether H2B de‐ubiquitination is also important for the induction of light‐regulated genes in a similar manner to the regulation of *FLOWERING LOCUS C* remains to be examined (Schmitz *et al*., 2009). Furthermore, it would be interesting to investigate whether plants possess a similar mechanism of sequential ubiquitination and de‐ubiquitination to activate gene expression as shown in yeast. Yeast H2B de‐ubiquitination is regulated by components of the SAGA (Spt‐Ada‐Gcn5 acetyltransferase) acetylation complex, such as UBP8 and GCN5 (Henry *et al*., 2003). Disruption of sequential ubiquitination and SAGA‐mediated de‐ubiquitination can affect the methylation status of H3K4 and H3K36 on gene loci (Henry *et al*., 2003). It would therefore be of great interest to examine the role of the Arabidopsis SAGA components, in particular GCN5, in modulating H2B de‐ubiquitination, H3K4 and H3K36 methylation and gene expression in response to light.

## Light shapes nuclear architecture

The nuclear organization of the eukaryotic genome is far from being random. Hierarchical and spatial distribution of chromosomes, chromatin domains and coregulated gene loci is tightly regulated through association with specific nuclear structures and protein complexes. Studies in yeast, fruit flies, humans and plants indicate that the position of a gene within the nucleus can influence its transcriptional potency (Gibcus & Dekker, 2013; Liu & Weigel, 2015; Randise‐Hinchliff & Brickner, 2016). Major developmental reprogramming events are usually accompanied by repositioning of gene loci towards the nuclear interior, the periphery or proximally to chromocentres depending on the species and the associated proteins that will determine the extent of gene activation or silencing (Takizawa *et al*., 2008; Feng *et al*., 2014; Bourbousse *et al*., 2015; Wang *et al*., 2015; Randise‐Hinchliff & Brickner, 2016; Rodriguez‐Granados *et al*., 2016). Thanks to recent developments in cytogenetic approaches, advanced imaging and whole‐genome biochemical technologies, there is an increasing amount of information demonstrating how light can trigger global nuclear changes in chromatin organization and gene topology (Tessadori *et al*., 2009; Feng *et al*., 2014; Bourbousse *et al*., 2015).

### Light‐induced changes in chromatin compaction

Changes in chromatin condensation allow developmental and ecological plasticity in plants grown in diverse habitats (Tessadori *et al*., 2009). Studies on natural Arabidopsis ecotypes have uncovered potential roles for the photoreceptors phyB and cry2 and histone deacetylases in light‐dependent chromatin compaction (Tessadori *et al*., 2007, 2009; van Zanten *et al*., 2010, 2012). The histone deacetylase HDA6 was identified by examining the degree of chromatin compaction in 21 Arabidopsis accessions exposed to various light intensities. Phenotypic screening of natural Arabidopsis populations coupled with quantitative trait locus (QTL) mapping revealed negative correlation between chromatin compaction and light intensity to which each accession was exposed to Tessadori *et al*. (2009). More specifically, the Arabidopsis accession Cape Verde Islands‐0 (Cvi‐0) showed the lowest heterochromatin index (HX), an indicator of low chromatin compaction. QTL analysis revealed a polymorphism in the *PHYB* locus and the *HDA6* promoter in Cvi‐0. Further studies on *hda6*,* phyB* and *cry2* mutants confirmed an active role in promoting chromatin reorganization (Table S1) (Tessadori *et al*., 2009). Low‐intensity blue light as well as low R : FR, known to trigger the shade avoidance syndrome, lead to reversible chromatin decompaction, which could be a prerequisite for major changes in gene expression that shape plant architecture to optimize growth (van Zanten *et al*., 2010, 2012).

### Photorelocation of gene loci

**Figure 4:**
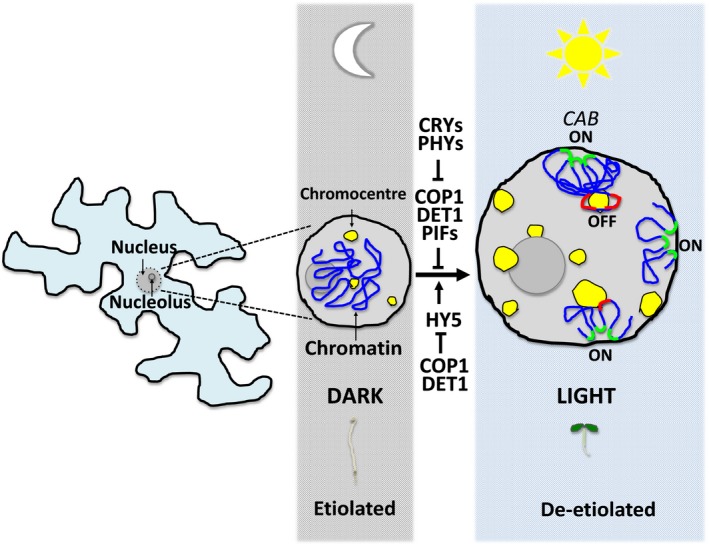
Light‐induced changes in cotyledon nuclear organization during photomorphogenesis. Light induces major changes in nuclear organization. During the transition from dark to light growth, there is a noticeable increase in the nuclear surface area accompanied by changes in chromatin compaction and the photorelocation of actively transcribed gene loci to the nuclear periphery. Light‐induced changes in nuclear architecture are triggered by the antagonistic and synergistic action of multiple photoreceptors and light signalling components. ON (green) and OFF (red) indicate active and inactive gene expression, respectively. CRYs, cryptochromes; PHYs, phytochromes; COP1, CONSTITUTIVE PHOTOMORPHOGENIC 1; DET1, DE‐ETIOLATED 1; PIFs, PHYTOCHROME INTERACTING FACTORS; HY5, ELONGATED HYPOCOTYL 5; *CAB*,*CHLOROPHYLL A/B‐BINDING*. The model is based on data shown by Tessadori *et al*. (2007, 2009), Feng *et al*. (2014) and Bourbousse *et al*. (2015).

The existence of light‐dependent gene repositioning in Arabidopsis was first demonstrated by revolutionary studies performed on photoreceptor and light signalling mutant backgrounds using a novel fluorescence *in situ* hybridization (padlock FISH) approach that enabled signal amplification (Feng *et al*., 2014). More specifically, the authors showed that light triggers the relocation of light‐induced loci, such as *CHLOROPHYLL A/B‐BINDING* (*CAB*), *RUBISCO SMALL SUBUNIT* (*RBCS*), *PLASTOCYANIN* (*PC*) and *GENOMES UNCOUPLED 5* (*GUN5*) to the periphery of the nucleus and that this repositioning directly correlates with an increase in their transcript abundance (Table S1; Fig. 4). (Feng *et al*., 2014). Cytogenetic and gene expression analysis have also revealed that phyA and phyB have a positive role in the repositioning and transcriptional activation of the aforementioned genes, whereas well‐established repressors of photomorphogenesis, COP1 and DET1, have the opposite effect (Feng *et al*., 2014). It would be of great interest to examine if photorelocation of light‐activated genes to the nuclear periphery overlaps with the localization of nuclear pore proteins (Nups).

Studies in yeast and metazoa have shown that actively transcribed genes reposition towards the nuclear envelope and interact with Nups, whereas inactive heterochromatin tends to localize in lamin‐associated domains (Gibcus & Dekker, 2013). Molecular characterization of the mechanism and the direct components mediating photorelocation and recruitment in specific nuclear topologies is essential. One possible hypothesis would entail that transcriptional regulators such as TFs and histone‐modifying enzymes would directly bind to common elements of coregulated gene loci and reposition them to specific nuclear domains to facilitate the rate of transcription. Similar mechanisms operate in yeast, where TFs and HDACs mediate gene repositioning in response to diverse stimuli (Randise‐Hinchliff & Brickner, 2016). Alternative mechanisms involving a decrease in the abundance of transcriptional corepressors or repressive histone marks may allow gene repositioning and regulation of gene expression (Towbin *et al*., 2012). Revolutionary imaging technologies using high‐throughput imaging mapping (HIPMap) in human cells have identified novel gene positioning factors ranging from chromatin remodelling and modifying enzymes, nuclear pore proteins and DNA replication‐associated factors (Shachar *et al*., 2015). HIPMap combined with RNA FISH could potentially examine the role of gene repositioning in gene expression not only at the single‐cell level but also at the single‐allele level.

Recent studies combining advanced immunofluorescence imaging and epigenomic approaches have provided groundbreaking information on the level of chromatin compaction before and during de‐etiolation (Bourbousse *et al*., 2015). The authors followed the topology of established histone marks and chromosomal regions during a time‐course from dark to light transition of etiolating seedlings and discovered not only an increase in total nuclear surface area, but also gradual repositioning of heavily methylated heterochromatin towards the chromocentres (also known to mark tightly packed chromatin in plants) (Table S1; Fig. 4) (Bourbousse *et al*., 2015). Wavelength‐specific illuminations and mutant genetic analysis showed that the cry2 blue light receptor is responsible for mediating major changes in nuclear architecture during de‐etiolation. Interestingly, studies on *det1* and *cop1* mutants revealed that these proteins are essential for maintaining chromatin decondensation in darkness (Bourbousse *et al*., 2015). Could light signalling components, such as COP1, PIFs and cry2, maintain chromatin flexibility and direct accessibility of transcriptional regulators to specific gene loci in the dark as a means of facilitating de‐etiolation upon light perception? To test this hypothesis, the next line of experiments would require functional verification and characterization of the molecular mechanism underlying light‐induced nuclear reorganization regulating gene expression and photomorphogenesis. Chromatin looping provides a great example whereby three‐dimensional (3D) chromatin interactions can affect the transcription of specific loci. Whether light‐induced chromatin reorganization mediates 3D changes to regulate the position or clustering of coactivated or corepressed loci in a similar manner as for the epigenetic silencing of the flowering repressor *FLOWERING LOWERING LOCUS C* (*FLC*) remains to be examined (Rosa *et al*., 2013; Sun *et al*., 2013).

### Photobodies: potential sites of transcriptional regulation?

In addition to changes in nuclear architecture, chromatin remodelling, reversible histone modifications and gene relocation mentioned in this review, light induces nuclear import and accumulation of signalling components that integrate light, hormone, circadian and stress pathways via synergistic or antagonistic interactions. Photoreceptors, transcriptional regulators and light signalling components cluster in nuclear microdomains, also known as photobodies (Van Buskirk *et al*., 2012).

The function of nuclear photobodies (NBs) still remains a mystery. There is an increasing number of functional studies suggesting that NBs could act as sites for protein degradation, transcriptional regulation or receptor desensitization (Al‐Sady *et al*., 2006; Chen *et al*., 2010; Zhang *et al*., 2013; Ni *et al*., 2014; Kaiserli *et al*., 2015; Klose *et al*., 2015; Qiu *et al*., 2015). The molecular mechanism driving the localization of protein complexes in NBs is still unclear, although PIF TFs seem to play a major role in phyB recruitment in photobodies (Al‐Sady *et al*., 2006; Pfeiffer *et al*., 2012). Furthermore, phyB is essential for recruiting transcriptional regulators such as TZP in NBs in response to R light (Kaiserli *et al*., 2015). Although TZP and phyB NB formation correlates with transcription, there is no direct evidence indicating that coregulated gene loci or newly transcribed mRNA populations cluster in these domains. However, it may not be coincidental that the majority of signalling components involved in mediating changes in nuclear architecture and gene repositioning in response to light have been observed to localize in NBs (phyB, cry2, PIFs, COP1). Whether the formation of NBs is a prerequisite or a consequence of chromatin reorganization remains to be investigated.

Do coregulated loci concentrate in nuclear vicinities enriched in transcriptional regulators and light signalling components? The existence of such nucleic acid and protein complexes, also known as ‘transcription factories’ is a possible hypothesis and their existence remains to be examined in plants. Determining the protein and genetic composition of specific NBs in a tissue‐ and developmental‐specific context is essential for providing more information on their role in light‐regulated nuclear organization. In addition, the role of post‐translational modifications, such as phosphorylation and sumoylation, in regulating these processes would be of equal interest. What is the driving force for gene repositioning and protein movement within the nucleus? Do photoreceptors have a direct role in regulating chromatin compaction by recruiting histone modifiers? These are just a few of the many questions to be answered in order to fully understand how light shapes the nucleus to allow major plant developmental transitions and acclimation responses to take place. Recent advances in single molecule sequencing and mass spectrometry could be applied to potentially answer these questions (Larance & Lamond, 2015; Anchel *et al*., 2016).

## Future perspectives: new technologies to shed light on photoregulated nuclear organization

Photoinduced nuclear reorganization has been discovered thanks to cytogenetic, biochemical and genetic studies on plants. Light coordinates changes in histone modifications and chromatin remodelling, leading to transcriptional changes in the expression of a number of genes involved in light and hormone signalling, metabolism, development and circadian regulation. Revolutionary technologies such as super‐resolution imaging and chromatin conformation capture (3C, 4C, Hi‐C) provide a powerful toolbox that will undoubtedly lead to exciting discoveries with regard to light‐regulated chromosomal territories and nuclear protein complexes (Betzig *et al*., 1991; Rust *et al*., 2006; Folling *et al*., 2008; Dekker *et al*., 2013). Hi‐C analysis in plants has already been used to study chromatin packing of the Arabidopsis genome at high resolution. Recent studies have revealed that Arabidopsis lacks canonical topologically associated domains (TADs) with high frequencies of contact among intrachromatin and ‘insulation’ from nearby chromatin regions (Wang *et al*., 2015). However, Arabidopsis does contain TAD boundary‐like and insulator‐like regions that are enriched in epigenetic modifications (Liu & Weigel, 2015; Wang *et al*., 2015). Investigating how light affects TAD‐like and insulator‐like regions would be of high priority.

Uncovering the mechanism by which light stimulates changes in plant nuclear architecture will provide invaluable knowledge that can be translated into diverse biological applications ranging from crop improvement to optogenetic regulation of stem cell differentiation.

## Supporting information

Please note: Wiley Blackwell are not responsible for the content or functionality of any Supporting Information supplied by the authors. Any queries (other than missing material) should be directed to the *New Phytologist* Central Office.

**Table S1** Overview of experimental evidence reporting light‐induced histone modifications and gene expression
