# Supplementary material for: Light behind the curtain: photoregulation of nuclear architecture and chromatin dynamics in plants
Source: New Phytol. 2016 Nov 4;212(4):908–19. doi: 10.1111/nph.14269 (PMC5111779; doi:10.1111/nph.14269)
Supplement: Supplementary file 1 — Table S1 Overview of experimental evidence reporting light‐induced histone modifications and gene expression [file NPH-212-908-s001.pdf]

New Phytologist **Supporting Information**

Article title: Light behind the curtain: photoregulation of nuclear architecture and chromatin dynamics in plants

Authors: Giorgio Perrella and Eirini Kaiserli

Acceptance date: 14 September 2016

The following Supporting Information is available for this article: Table S1

Table S1 List of selected studies that analysed histone modifications and gene expression in response to light. The table includes the species and genotype, type of histone modifications and genes examined, plant tissue, developmental stage and the light conditions used.

**Species/ Ecotype/ Genotype**	**Histone modifications tested**	**Genes tested**	**Plant tissue/developm ental stage**	**Light source**	**Reference**
Pisum sativum WT	H3Ac, H4Ac	*PETe*	Shoots/etiolation	White light	Chua *et al.*, 2003
A. thaliana Ws *haf2-1,* Col-0 *haf2-2,* Ler *hy1, hy5*	H3K9K14Ac, H4K5K8K12K16	*CAB2, RBCS*	Hypocotyl/de-etiolation	White light, Red light, Far-red light, Blue light	Bertrand *et al.*, 2005
A. thaliana Ws *taf-1, gcn5-1, athd-1,* Ler *hy5*	H3K9K14Ac, H3K27Ac, H4K5K8K12K16Ac	*CAB2, RBCS, IAA3, HY5*	Hypocotyl/de-etiolation	Red light, Far-red light	Benhamed *et al.*, 2006
A. thaliana phyB-9, phyA Col-0, *athd-1, taf1* Ws	H3K4me2, H3K4me3, H3K9/14ac,H3K27ac, H3K36me3, H3K9me2, H3K9me3, H3K27me3 H4K5K8K12K16Ac	*PHYA*	Whole plants/ 2 wk old	White light	Jang *et al.*, 2011
A. thaliana Col-0, Ler, cry1-304-cry2-1 phyA phyB phyE, phyB phyD phyE, cop1-4, det1-1, hy5-215	H3K4me3, H3K9ac, H3K14Ac H3K9me2, H3K27me3	*At2g34430, At3g54890, At5g38430 , At5g62300, At2g37130,At2g42790*	Seedlings/ 6 d old	White, Blue, red, far-red light	Guo *et al.*, 2008;
A.thaliana, uvr8-1, hy5/hyh, gcn5, taf1, hac5, hd1, fve,Ler, ws, Col-0	H3K9/K14 diacetylation, H3K56ac H2Bub ,H3K4me3, H3K36me3	*MYB12, CRYD, ELIP1, CHS, HYH, HY5, WRKY30*	3 wk old, leaf tissue, 5 d old, hypocotyl elongation assay	White light -/+ UV-B	Brown *et al.*, 2005; Cloix *et al.*, 2008; Velanis *et al.*, 2016

Zea mays B73, Confite Punen˜o, Mishca, W23b, RNAi chc101, mbd101	H3Ac, H4Ac, H4	*NFC102, TH-LIKE*	Leaves	White light -/+ UV-B	Casati *et al.*, 2008
Zea mays B73, Amarillo, Mishca	H3K9me2, H3K27me3, H3	*ZmP1*	Whole plants/ 5 wk old	White light -/+ UV-B	Rius *et al.*, 2016
A. thaliana, Col-0	H3K9ac, H3K9me3, H3K27ac, and H3K27me3	*Genome wide*	Seedlings/ 6 d old	White light	Charron *et al.*, 2009
A. thaliana Col-0, sdg8-5	H3K36me3, H3K4me3	*Genome wide*	Whole plants/ 3 wk old	White light	Li *et al.*, 2015
A. thaliana jmj20-1 and jmj22-1, som-1, som-2, phyB-9, pil5-1 Col-0	H3K9me3, H3K4me3,H4R3me2	*GA3OX1, GA3OX2*	Seeds	Far-red light	Cho *et al.*, 2012
A. thaliana Col-0, epp1-1 pkl-1, epp1-2 epp1-3, exp2-1 , dwf4-11 , phyA-211 phyB-9 cry1-304 hy5-215 (Oyama et al., 1997), hyh, cop1-4, pkr1-1, pkr2-2, chr5-1	H3K27me3, H3	*HY5, EXT3, EXP2, XTH17, XTR6, DWF4, and IAA19, PRE1, TCH4, At4g02330, ACT2*	Hypocotyl/ de-etiolation	White light, Red light, Far-red light, Blue light	Jing *et al.*, 2013; Zhang *et al.*, 2014
A. thaliana hub1-1 Ler, hub1-3 , hub1-5, hub1-4, and hub2-2 Col-0	H2Bub ,H3K4me3, H3K36me3	*HCF173, TZP, GIGANTEA, RCC1 and PsbP-1*	Hypocotyl/ de-etiolation	White light	Bourbousse *et al.*, 2012

A. thaliana Ler, Cvi, Se-0, Ts-1, Sf-1, Lan-0, Be-0, Chi-1, Ws-2, Can-0, C24, Hel-1, Hir-1, Kas-2, Knox-10, Kond, Mt-0, Nd-1, Pak-1, (RIL, NIL), phyB-9, hda6, Sha, RLD-1, 35S:PHYB/phy b-9/Ler, 5S:PHYB/phyb -9/Cvi	Heterochromatin Index measured based on DAPI stained chromocenters. FISH on centromeric 180 bp, subtelomeric 45S rDNA repeats, pericentromeric sequences 5S rDNA, transposon-rich BAC F28D6. Immunofluorescence: histones, H3K9Me2, 5-Methylcytosine		Mesophyll interphase nuclei from young rosette leaves.	White light (various fluence rates 50–600 µmol m^{-2} s^{-1})	Tessadori, *et al*., 2009
A. thaliana: phot1-101, phot2-5, phot1-101 phot2-5/Ws-2/Ler, cry1 cry2 phot1 phot2/ Ws-2/Ler, cry1/Col-0, cry2-1 /Col-0, cry2 (fha1 in Ler), cry1 cry2 (hy4 fha1/Ler, 35S:CRY2, phyb9/ Col; phya201, phya-201 phyb5, hy2, phy-abde, phyb-1 phyd-2, phyb phye-1, phyb5 cry1-1, phyb5 cry2	Heterochromatin Index measured based on DAPI stained chromocenters. FISH on centromeric 180 bp, transposon-rich BAC F28D6.		Mesophyll interphase nuclei from young rosette leaves.	White light, Low R:FR, flowering transition during long day (18 h L/6 h D) photoperiod.	van Zanten *et al*., 2010
A. thaliana, Col-0, phyB-9, phyA-211, hy5, cop1-4, det1-1pifq, 35S:YHB,	DAPI stained chromocenters, padlock FISH: centromeric 180-bp repeats, IF: CENH3	*UBQ11, CAB, RBCS1A, PC, GUN5, PP2A*	Cotyledons (4 d old), mesophyll, epidermal, vascular, guard, meristem cells.	Dark, red light (time-course) far-red light.	Feng *et al*., 2013

A. thaliana, Col-0, det1-1, cop1-4 , hy5, cry1-304, cry2-1, phyB-9, phyA-211, 35S::H2B-YFP ,cop1/hy5	Heterochromatin Index measured based on DAPI stained chromocenters. FISH on centromeric 180 bp, transposon-rich BAC F28D6. Immunofluorescence: H3K9Me2, 5-Methylcytosine, H3K4me2, Fluorescent reporter: H2B-YFP.		Cotyledons	Dark, red, far-red, blue, white light	Bourbousse *et al*., 2015

References

Benhamed M, Bertrand C, Servet C, Zhou DX. 2006. Arabidopsis GCN5, HD1, and TAF1/HAF2 interact to regulate histone acetylation required for light-responsive gene expression. *Plant Cell* **18**: 2893–2903.

Bertrand C, Benhamed M, Li YF, Ayadi M, Lemonnier G, Renou JP, Delarue M, Zhou DX. 2005. Arabidopsis HAF2 gene encoding TATA-binding protein (TBP)-associated factor TAF1, is required to integrate light signals to regulate gene expression and growth. *J Biol Chem* **280**: 1465–1473.

Bourbousse C, Ahmed I, Roudier F, Zabulon G, Blondet E, Balzergue S, Colot V, Bowler C, Barneche F. 2012. Histone H2B monoubiquitination facilitates the rapid modulation of gene expression during Arabidopsis photomorphogenesis. *PLoS Genet* **8**: e1002825.

Bourbousse C, Mestiri I, Zabulon G, Bourge M, Formiggini F, Koini MA, Brown SC, Fransz P, Bowler C, Barneche F. 2015. Light signaling controls nuclear architecture reorganization during seedling establishment. *Proc Natl Acad Sci U S A* **112**: E2836–2844.

Brown BA, Cloix C, Jiang GH, Kaiserli E, Herzyk P, Kliebenstein DJ, Jenkins GI. 2005. A UV-B-specific signaling component orchestrates plant UV protection. *Proc Natl Acad Sci U S A* **102**: 18225–18230.

Casati P, Campi M, Chu F, Suzuki N, Maltby D, Guan S, Burlingame AL, Walbot V. 2008. Histone acetylation and chromatin remodeling are required for UV-B-dependent transcriptional activation of regulated genes in maize. *Plant Cell* **20**: 827–842.

Charron JB, He H, Elling AA, Deng XW. 2009. Dynamic landscapes of four histone modifications during deetiolation in Arabidopsis. *Plant Cell* **21**: 3732–3748.

Cho JN, Ryu JY, Jeong YM, Park J, Song JJ, Amasino RM, Noh B, Noh YS. 2012. Control of seed germination by light-induced histone arginine demethylation activity. *Dev Cell* **22**: 736–748.

Chua YL, Watson LA, Gray JC. 2003. The transcriptional enhancer of the pea plastocyanin gene associates with the nuclear matrix and regulates gene expression through histone acetylation. *Plant Cell* **15**: 1468–1479.

Cloix C, Jenkins GI. 2008. Interaction of the Arabidopsis UV-B-specific signaling component UVR8 with chromatin. *Mol Plant* **1**: 118–128.

Feng CM, Qiu Y, Van Buskirk EK, Yang EJ, Chen M. 2014. Light-regulated gene repositioning in Arabidopsis. *Nat Commun* **5**: 3027.

Guo L, Zhou J, Elling AA, Charron JB, Deng XW. 2008. Histone modifications and expression of light-regulated genes in Arabidopsis are cooperatively influenced by changing light conditions. *Plant Physiol* **147**: 2070–2083.

Jang IC, Chung PJ, Hemmes H, Jung C, Chua NH. 2011. Rapid and reversible light-mediated chromatin modifications of Arabidopsis *PHYTOCHROME A* locus. *Plant Cell* **23**: 459–470.

Jing Y, Zhang D, Wang X, Tang W, Wang W, Huai J, Xu G, Chen D, Li Y, Lin R. 2013. Arabidopsis chromatin remodeling factor PICKLE interacts with transcription factor HY5 to regulate hypocotyl cell elongation. *Plant Cell* **25**: 242–256.

Li Y, Mukherjee I, Thum KE, Tanurdzic M, Katari MS, Obertello M, Edwards MB, McCombie WR, Martienssen RA, Coruzzi GM. 2015. The histone methyltransferase SDG8 mediates the epigenetic modification of light and carbon responsive genes in plants. *Genome Biol* **16**: 79.

Rius SP, Emiliani J, Casati P. 2016. P1 Epigenetic Regulation in Leaves of High Altitude Maize Landraces: Effect of UV-B Radiation. *Front Plant Sci* **7**: 523.

Tessadori F, van Zanten M, Pavlova P, Clifton R, Pontvianne F, Snoek LB, Millenaar FF, Schulkes RK, van Driel R, Voesenek LA *et al*. 2009. Phytochrome B and histone deacetylase 6 control light-induced chromatin compaction in *Arabidopsis thaliana*. *PLoS Genet* **5**(9): e1000638.

van Zanten M, Tessadori F, Bossen L, Peeters AJ, Fransz P. 2010. Large-scale chromatin de-compaction induced by low light is not accompanied by nucleosomal displacement. *Plant Signal Behav* **5**: 1677–1678.

Velanis CN, Herzyk P, Jenkins GI. 2016. Regulation of transcription by the Arabidopsis UVR8 photoreceptor involves a specific histone modification. *Plant Mol Biol*. doi: 10.1007/s11103-016-0522-3.

Zhang D, Jing Y, Jiang Z, Lin R. 2014. The Chromatin-Remodeling Factor PICKLE Integrates Brassinosteroid and Gibberellin Signaling during Skotomorphogenic Growth in Arabidopsis. *Plant Cell* **26**: 2472–2485.
